# Qiang-Xin 1 Formula Prevents Sepsis-Induced Apoptosis in Murine Cardiomyocytes by Suppressing Endoplasmic Reticulum- and Mitochondria-Associated Pathways

**DOI:** 10.3389/fphar.2018.00818

**Published:** 2018-07-30

**Authors:** Xiaolong Xu, Qingquan Liu, Shasha He, Jingxia Zhao, Ning Wang, Xuyang Han, Yuhong Guo

**Affiliations:** ^1^Beijing Hospital of Traditional Chinese Medicine, Affiliated with Capital Medical University, Beijing, China; ^2^Beijing Institute of Traditional Chinese Medicine, Beijing, China; ^3^Beijing Key Laboratory of Basic Research with Traditional Chinese Medicine on Infectious Diseases, Beijing, China

**Keywords:** apoptosis, ER stress, mitochondria, murine, sepsis, traditional Chinese medicine

## Abstract

Sepsis is reported to be an unusual systemic reaction to infection, accompanied by multiple-organ failure. Sepsis-induced cardiomyopathy (SIC), defined as damages and dysfunction of the heart, is essential in the pathogenesis of sepsis. Traditional Chinese formula, which has long been used to improve the situation of patients through multitarget regulation, is now gradually being used as complementary therapy. The present study aimed to investigate the effect of Qiang-Xin 1 (QX1) formula, a traditional Chinese herbal medicine designed for cardiac dysfunction, on cecal ligation puncture (CLP)-induced heart damage and its underlying mechanisms in mice. Survival test first showed that an oral administration of QX1 formula significantly increased the 7-days survival of septic mice from 22 to 40%. By estimating the secretion of serum cytokines, QX1 treatment dramatically inhibited the excessive production of interleukin-1β and tumor necrosis factor-α. Immunohistochemical staining illustrated that the expression of c-Jun N-terminal kinase, caspase-12, and high-mobility group box 1 was downregulated in cardiomyocytes of the QX1-treated group compared with that of the CLP surgery group. Western blotting confirmed that the activation of essential caspase family members, such as caspase-3, caspase-9, and caspase-12, was prohibited by treatment with QX1. Moreover, the abnormal expression of key regulators of endoplasmic reticulum (ER) and mitochondria-associated apoptosis in cardiomyocytes of septic mice, including CHOP, GRP78, Cyt-c, Bcl-2, Bcl-X_L_, and Bax, was effectively reversed by treatment with QX1 formula. This study provided a new insight into the role of QX1 formula in heart damage and potential complementary therapeutic effect of traditional Chinese medicine on sepsis.

## Introduction

Sepsis is newly defined as an unusual systemic reaction to severe infection and recognized as one of the deadliest menace in intensive care unit (ICU) (Shankar-Hari et al., 2016). Approximately, 26 million people worldwide are affected by this life-threatening disease every year (Teng et al., 2017). According to established studies, the pathophysiology of sepsis contains hyperinflammatory response, immune dysfunction, organ failure, and impaired tissue perfusion (Horak et al., 2017). Over the past decades, early recognition and management of excessive inflammatory reactions, primarily using anti-inflammatory therapy, have been the focus; however, the morbidity and mortality of septic patients remain high (Dellinger, 2015), prompting the requirement of more complementary investigations.

The dysfunction of vital organs, including heart, lung, and liver, is not only a typical pathological characteristic of sepsis but also an essential cause of high mortality (Greco et al., 2017). The heart is an important pump organ that may also suffer serious damages in sepsis. Sepsis-induced cardiomyopathy (SIC) is characterized as systolic and diastolic, but reversible, dysfunction of both sides of the heart. It is a syndrome induced by myocardial depressants from the source of infection in septic patients (Antonucci et al., 2014). Nearly, 60% septic patients exhibit signs of myocardial depression and damages (Vieillard-Baron et al., 2008). Although several studies have pointed out that the immunosuppression occurring in late stages of sepsis is the primary reason for mortality, the cytokine storm and cardiac dysfunction occurring in the early onset of sepsis are also essential causes of organ dysfunction and mortality of septic patients (Hotchkiss et al., 2013). Septic cardiac dysfunction has been associated with excessive production of cytokines, including interleukin-6 (IL-6), tumor necrosis factor-α (TNF-α), and high-mobility group box-1 (HMGB1), which also contribute to myocyte apoptosis and damages (Buerke et al., 2008; Wang et al., 2014; Zhang et al., 2015; Han et al., 2017).

Endoplasmic reticulum (ER) and mitochondria are essential to apoptosis and thus contribute to the development of pathological cardiomyopathy and heart failure (Hsu et al., 2017; Misaka et al., 2017; Yao et al., 2017). ER is the primary site of protein processing, which can be affected by kinds of stress and lead to accumulation of unfolded or misfolded proteins. ER stress sensors activate downstream factors, including C/EBP homologous protein (CHOP), c-Jun N-terminal kinase (JNK), and caspase12, and finally contribute to the generation of unhealthy myocytes, followed by a cardiac failure (Ron and Walter, 2007; Minamino and Kitakaze, 2010; Cominacini et al., 2015; Wang et al., 2017). Mitochondria-mediated apoptosis is another main cause of heart failure (Steiner and Lang, 2017; Gao et al., 2018). Bcl-2 family members are vital in mitochondrial apoptotic pathways, acting as pro-apoptotic (Bax) and anti-apoptotic (Bcl-2 and Bcl-X_L_) parts (Kalkavan and Green, 2018).

Qiang-Xin 1 (QX1) is a traditional Chinese herbal formula designed by Prof. Lijuan Huang, a national celebrated traditional Chinese medicine expert. It has been used for clinical treatment of heart failure for more than 30 years and provided favorable effect. In a recent clinical study, QX1 treatment showed promising therapeutic effects on septic patients as a complementary therapy (data unpublished), indicating that QX1 might provide a better outcome of septic patients by improving the function of heart. Animal experiments partly demonstrated that QX1 protected myocytes from apoptosis in septic mice. Therefore, this study aimed to assess the compatibility effects and characteristic of QX1 on sepsis-induced heart damages in mice and gain insights into its regulatory role in ER- and mitochondria-associated apoptosis.

## Materials and Methods

### Preparation of QX1 Formula

QX1 formula, consists of Shui hong hua zi (*Polygonum orientale* L.) 30 g (3/8), Huang qi [*Astragalus membranaceus (Fisch.) Bge. var. mongholicus (Bge.) Hsiao*] 30 g (3/8), Fu ling 20 g [*Poria cocos (Schw.) Wolf*) (2/8), Dan shen (*Salvia miltiorrhiza Bge.*) 20 g (1/8), and Wu wei zi (*Schisandra chinensis (Turcz.) Baill.*] 10 g (1/8), was decocted and provided by TCM Pharmacy of Beijing hospital of traditional Chinese medicine (Detailed information of herbs in Supplementary Data Sheet S1). Briefly, 110 g crude drugs of QX1 formula were soaked and decocted in 400 mL pure water for 30 min. Then the water decoction was concentrated to 110 mL, and the final dosage of crude drugs was 1 g/mL. The dosages of QX1 formula used for this study, indicated as low dose, medium dose, and high dose, were 0.25, 0.5, and 1 g/kg⋅bw, respectively.

### Reagents

Hematoxylin and eosin (H&E) for HE staining were purchased from Sigma-Aldrich Chemical (St. Louis, MO, United States). TRIzol^®^ reagent were purchased from Gibco (Grand Island, NY, United States). Bicinchoninic acid (BCA) protein assay kit was purchased from Pierce (Rockford, IL, United States). The ELISA kits for IL-1β, TNF-α, and IL-10 were purchased from Cusabio (Wuhan, China). Antibodies for β-actin, Bcl-2, Bcl-x_L_, Bax, caspase-3, caspase-7, caspase-9, caspase-12, cleaved caspase-3, cleaved caspase-7, cleaved caspase-9, cleaved caspase-12, CHOP, GRP78, GRP94, JNK, p-JNK, and HMGB1 were purchased from Cell Signaling Technology (Danvers, MA, United States). The goat anti-mouse antibody was purchased from Li-cdr Odyssye^®^ (Lincoln, NE, United States).

### UPLC–MS/MS Analysis of QX1 Formula

A Waters ultra-high performance liquid chromatography-tandem mass spectrometry (UPLC–MS/MS) spectrometer equipped with a HESI-II probe was employed. The pos and neg HESI-II spray voltages were 3.7 and 3.5 kV, respectively, and the heated vaporizer temperature was 300°C. Both the sheath gas and the auxiliary gas were nitrogen. The collision gas was also nitrogen at a pressure of 1.5 mTorr. Data were collected and processed with Waters Masslynx 4.1 and UNIFI scientific information system. Quantitative analysis of dominating components was performed by employing Waters Acquity UPLC H-Class system. Acquity UPLC HSS T3 column (2.1 mm × 100 mm, 1.8 μm) was selected for analyses. The mobile phase was compose of A (water, 0.1% formic acid, v/v) and B (methanol, 0.1% formic acid, v/v), with a linear gradient elution. The flow rate was set to 0.3 mL/min and the column temperature was maintained at 45°C. Data were collected and analyzed by using Waters Masslynx 4.1 system.

### Establishment of Murine CLP Model

One hundred and twenty male BALB/c mice, 8-week old (18–22 g), were randomly divided into six treatment groups. Murine CLP model was established following protocol as described before (Hu et al., 2012). Briefly, mice subjected to CLP surgery were fasted for 24 h with water permitted before operation. Anesthetized mice were then opened in abdominal cavity and punctured once with a 5-gauge needle in cecum (1 cm from ileocecal junction). Finally, cecum was cautiously relocated into abdomen and incision was sewed up. Mice were injected with 1 mL 37°C normal saline subcutaneously and fasted for another 12 h. In our hands, the 48 h mortality of this model is about 80%.

### Enzyme-Linked Immunosorbent Assay (ELISA)

To detect the levels of released cytokines in serum, including IL-1β, TNF-α, and IL-10, blood sample from each group was rest at room temperature for 2 h and then centrifuged at 1,000 *g* for 20 min to obtain serum. The serum samples were then collected and immediately assayed. All protocols were performed as per the manufacturer’s instructions.

### Microarray-Based Transcriptional Profiling and Bioinformatic Analysis

Illumina HiSeq^TM^ 2000 was employed for RNA sequencing. Total RNA samples were first treated with DNase I to degrade any possible DNA contamination. Then the mRNAs are enriched by the oligo (dT) magnetic beads. cDNA was synthesized and sequencing was performed. Further bioinformatic analysis, including KEGG pathway enrichment analysis, Gene Ontology enrichment analysis, cluster analysis were employed to understand underlying mechanisms.

### H&E Staining and Immunohistochemistry

For H&E staining, heart tissues isolated from each group were fixed in 10% formalin and then embedded in paraffin. Fixed heart tissues (5 μM) were sectioned and stained with H&E. For immunohistochemistry, sections were incubated with 3% H_2_O_2_, blocking serum, and indicated antibodies for 2 h at 37°C. The sections were then washed using PBS and secondary antibodies for another 30 min. After washing with PBS for another three times, the sections were incubated with HRP-conjugated buffer and visualized using DAB buffer.

### Echocardiography

Mice were lightly anesthetized with 1% inhaled isoflurane and then estimated using RMV 707B High Resolution Imaging System (VisualSonics, Inc., Canada) equipped with a 30 MHz ultrasound probe. The ejection fraction (EF %) and fractional shortening (FS %) of left ventricle were calculated from M-mode tracing to reflect systolic function.

### Western Blotting

In brief, the proteins obtained from heart tissues were homogenized in PBS and centrifuged at 16,000 rpm for 10 min in 4°C. Supernatants were collected and quantified using BCA assay kit (Pierce, Rockford, IL, United States). Protein bands were separated using SDS-PAGE and blotted to nitrocellulose membranes (Pierce, IL, United States), and then incubated for 24 h at 4°C with specific antibodies.

## Results

### Identification of Major Components of QX1 Formula

**FIGURE 1:**
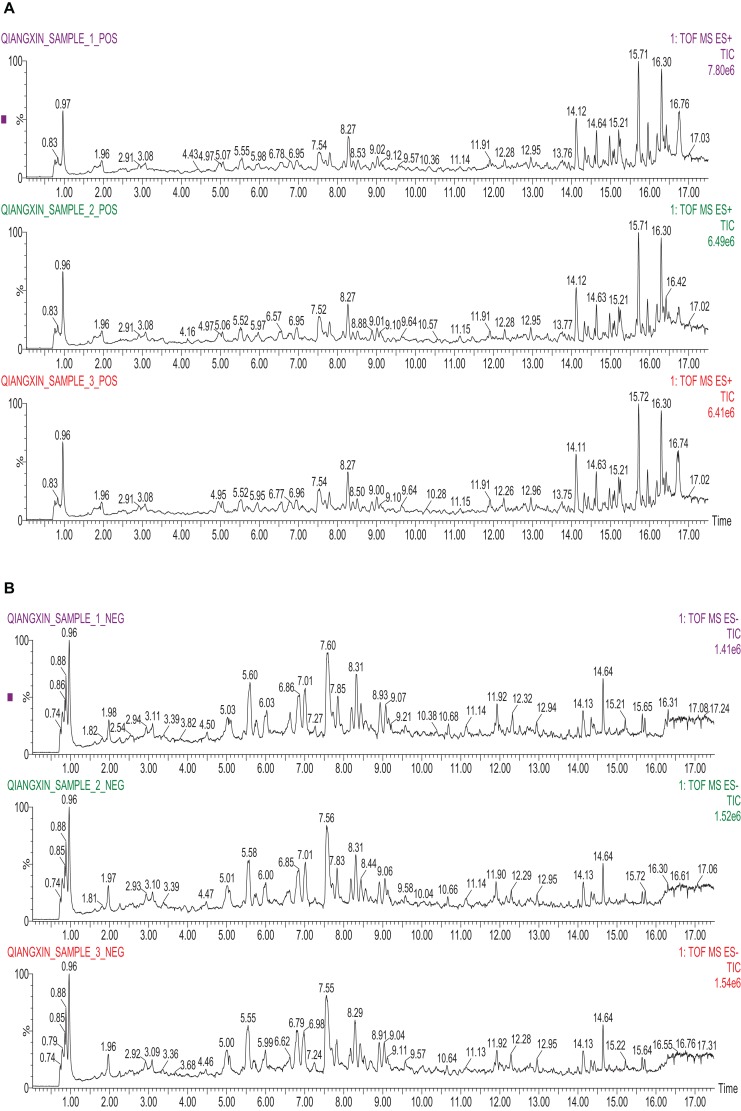
Identification of major components of QX1 formula. Three parallel samples of QX1 formula were examined using UPLC–MS/MS. Data were collected and proceeded by software Masslynx 4.1. The positive **(A)** and negative **(B)** ion chromatograms of QX1 formula were shown as indicated.

This study employed UPLC–MS/MS detection to investigate the ingredients and evaluate the repeatability and stability of QX1 formula. As shown in **Figure 1**, the total ion chromatogram of three parallel samples illustrated the composition and percentage of ingredients in the QX1 formula, such as gallic acid, cryptotanshinone, tanshinone IIA, texifolin, danshensu-sodium, and so forth (details in Supplementary Data Sheet S2). The contents of dominating compounds were further listed in Supplementary Table S1. Moreover, this study based on the characteristic peaks found that the QX1 formula showed high repeatability and stability according to coefficient correlation.

### QX1 Treatment Improved the Survival Rate of Cecal Ligation Puncture Mice

**FIGURE 2:**
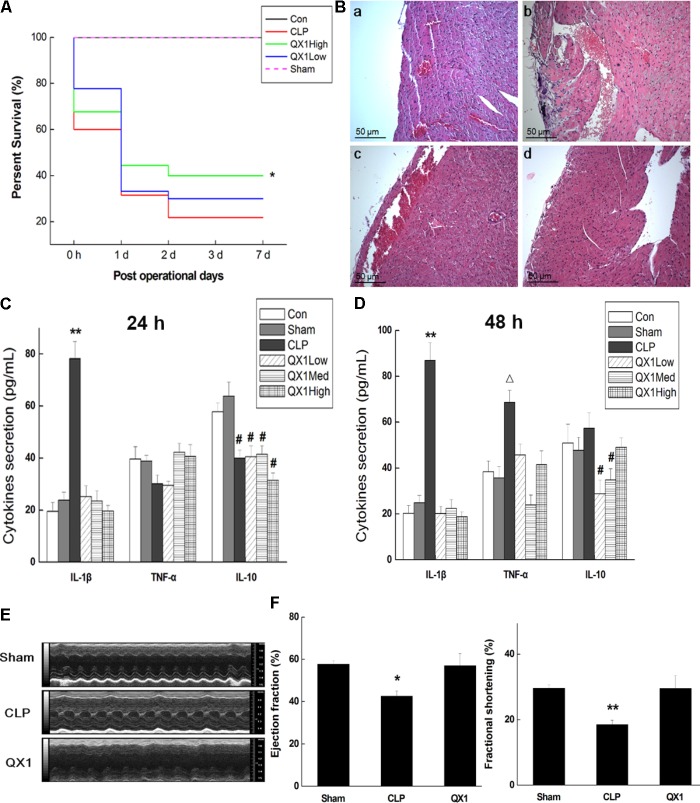
QX1 treatment improves survival rate of CLP mice. QX1 formula was orally administrated 2 h after surgery. The dosages of QX1 formula used for this study, indicated as low dose, medium dose, and high dose, were 0.25, 0.5, and 1 g/kg⋅bw, respectively. **(A)** Kaplan–Meier curves for survival rate. Thirty mice of each treatment group were used for comparison. The observation of mortality continued for 7 days, ^∗^*p* < 0.05 indicates significant difference compared with CLP surgery group. **(B)** H&E staining of heart tissue in each group. **(a)** Control group, **(b)** CLP surgery group, **(c)** QX1 high dose group, **(d)** sham group. **(C)** ELISA detection on expression of serum cytokines (24 h after surgery). ^∗∗^*p* < 0.01 and ^#^*p* < 0.05 indicate significant differences compared with sham group. **(D)** ELISA detection on expression of serum cytokines (48 h after surgery). ^∗∗^*p* < 0.01, ^Δ^*p* < 0.05 and ^#^*p* < 0.05 indicate significant differences compared with sham group. **(E)** Echocardiography was employed to evaluate changes in heart function of mice in sham group, CLP group, and high dose QX1 treatment group. **(F)** Evaluation of the ejection fraction (EF %) and fractional shortening (FS %) of mice in sham group, CLP group, and high dose QX1 treatment group.

This study initially explored whether oral administration of QX1 possessed a survival advantage to septic mice. As shown in **Figure 2A**, survival data proved that QX1 treatment significantly reduced the death rate of mice subjected to CLP surgery throughout 7 days of observation. Histological evaluation of myocardium revealed that CLP surgery markedly promoted edema, hyperplasia, and inflammatory infiltrate in cardiomyocytes. QX1 treatment bettered the situation of heart tissue by partially prohibiting inflammatory infiltrate and edema. Degeneration and necrosis in myocardial cells at the edge in CLP mice were also alleviated by QX1 treatment (**Figure 2B**). The results based on ELISA further demonstrated that QX1 treatment notably suppressed serum IL-1β secretion at 24 and 48 h after CLP surgery (*p* < 0.05) and decreased TNF-α production at 48 h (*p* < 0.05). However, QX1 showed the limited beneficial effect on regulating IL-10 generation (**Figures 2C,D**). 7 days secretion of cytokines of each group showed no significant differences (details in Supplementary Table S2). Echocardiography illustrated that CLP surgery (48 h) resulted in remarked reduction of ejection fraction (*p* < 0.05) and fraction shortening (*p* < 0.01) in left ventricle of mice, which were dramatically reversed by treatment of high dose QX1 formula (**Figures 2E,F**).

### Microarray-Based Genomic Detection and Bioinformatics Analysis on Myocytes of QX1-Treated Septic Mice

**FIGURE 3:**
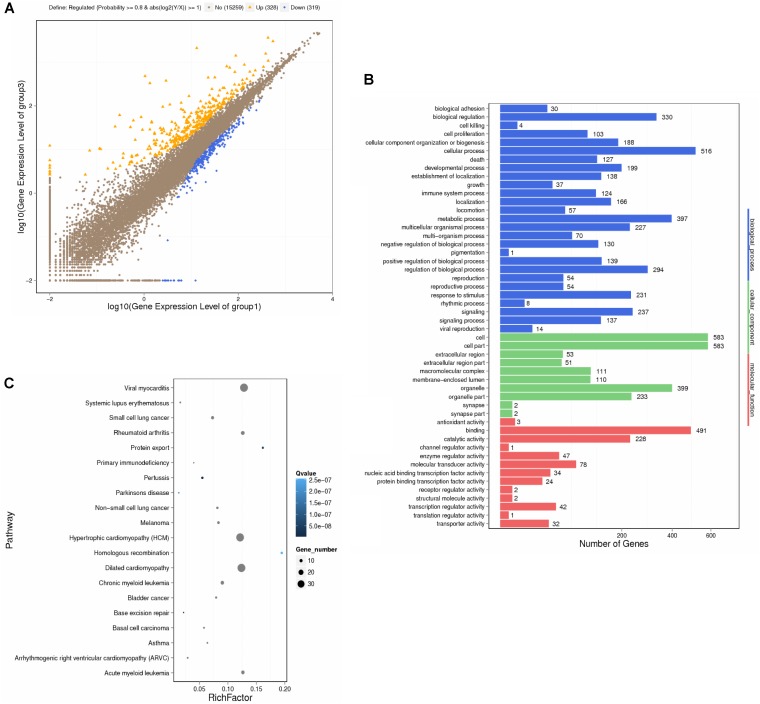
Transcriptional profiling and bioinformatic analysis of cardiomyocytes in mice. The screening and identification of differential genes were performed between QX1 treated group (high dose, 1 g/kg⋅bw) and CLP surgery group (48 h). **(A)** Scatter plots of genes in each pairwise. X- axis and Y- axis both present the log2 value of all gene expression. Blue spots indicate down-regulation genes (*p* < 0.05), orange spots indicate up-regulation genes (*p* < 0.05), and brown spots indicate non-regulation genes (*p* > 0.05). **(B)** Gene ontology (GO) analysis of differential genes. WEGO software were employed to perform GO functional classifications, including biological process, cellular component, and molecular function. **(C)** Pathway enrichment analysis of differential genes. The KEGG database was employed to perform pathway enrichment analysis. Pathways with *Q* ≤ 0.05 were identified as enriched pathways.

**FIGURE 4:**
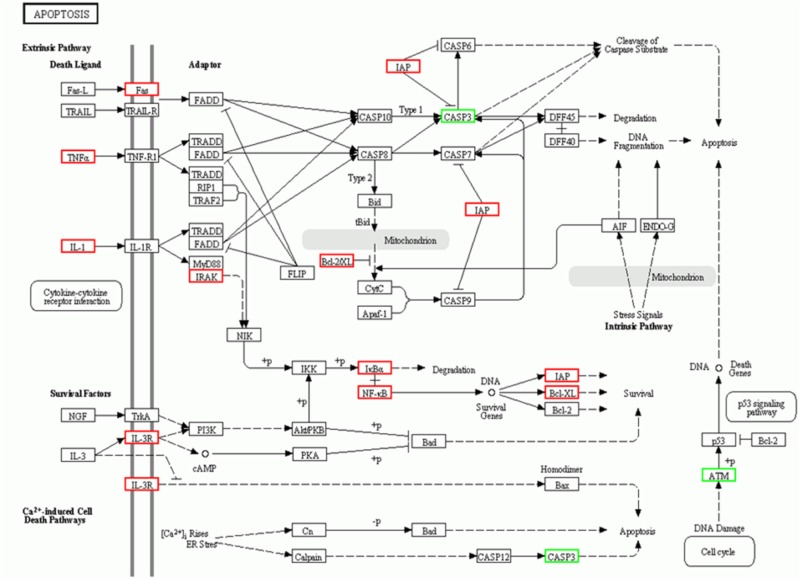
KEGG signaling pathways of apoptosis in cardiomyocytes. The analysis of differential genes were performed between QX1 treated group (high dose, 1 g/kg⋅bw) and CLP surgery group. Up-regulated genes (*p* < 0.05) were marked with red borders while down-regulated genes (*p* < 0.05) with green borders.

This study employed microarray-based investigation to detect the gene expression profiles so as to better understand the systematic regulatory properties of QX1 on myocytes of CLP mice. The data illustrated that 328 genes were significantly upregulated while 319 downregulated in myocytes of mice treated with QX1 formula, compared with the CLP surgery group. Most of these different genes were involved in biological processes including cellular process, metabolic process, biological regulation, and so forth (**Figures 3A,B**). The pathway enrichment analysis further revealed differentially expressed genes in myocytes in diseases such as myocarditis, hypertrophic cardiomyopathy (HCM), and dilated cardiomyopathy (**Figure 3C**). Moreover, Kyoto Encyclopedia of Genes and Genomes (KEGG) analysis showed that many genes encoding vital regulators in anti-apoptosis pathways were activated in myocytes by QX1 treatment, including Bcl2, Bcl-X_L_, nuclear factor of kappa light polypeptide gene enhancer in B-cells inhibitor alpha (IκBα), nuclear factor kappa light chain enhancer of activated B cells (NF-κB), and inhibitor of apoptosis (IAP), compared with septic mice. Meanwhile, this study found that pro-apoptotic genes, such as caspase-3 and ataxia telangiectasia mutated (ATM), were downregulated by treatment with QX1 (**Figure 4**).

### QX1 Treatment Prevented Apoptosis in Myocytes of Septic Mice

**FIGURE 5:**
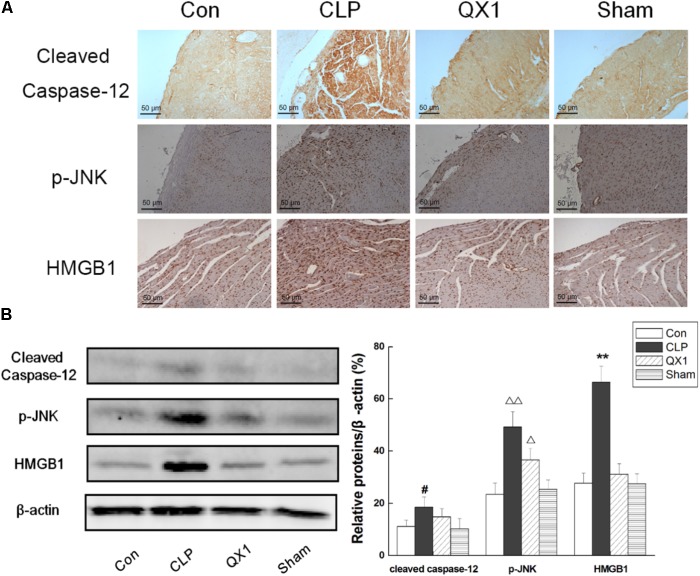
**(A)** Immunohistochemical staining of heart tissue for the expression of caspase-12, p-JNK, and HMGB1 in representative treatment group. High dose of QX1 formula (1 g/kgbw) was used for detection and all samples were harvested 48 h after surgery. **(B)** QX1 inhibits expression of cleaved caspase-12, p-JNK, and HMGB1. Data represent the mean ± SD of three independent experiments and differences between mean values were assessed by one-way ANOVA. ^∗∗^*p* < 0.01, ^Δ^ *p* < 0.05, ^ΔΔ^ *p* < 0.01, and ^#^*p* < 0.05 indicate significant differences compared with control group.

**FIGURE 6:**
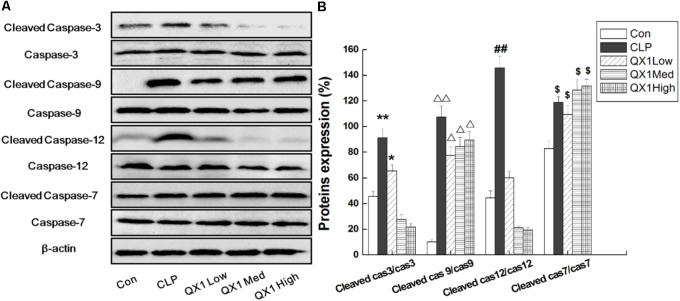
**(A)**QX1 treatment prevents caspase family members activation in cardiomyocytes. QX1 formula of three dosages (indicated as low dose, medium dose, and high dose, were 0.25, 0.5, and 1 g/kgbw, respectively.) were orally administrated 2 h after surgery and heart tissue were obtained 48 h after CLP surgery for western blotting detection. **(B)** Statistical analysis of indicated proteins expression. Data represent the mean ± SD of three independent experiments and differences between mean values were assessed by one-way ANOVA. ^∗^*p* < 0.05, ^∗∗^*p* < 0.01, ^Δ^ *p* < 0.05, ^ΔΔ^ *p* < 0.01, ^##^*p* < 0.01, ^$^*p* < 0.05 indicate significant differences compared with control group.

Since genomic analysis hinted that the beneficial actions of QX1 on septic mice survival were probably caused by its regulatory effect on apoptotic signaling in myocytes, this study determined the anti-apoptotic effect of QX1 on heart tissue of septic mice. Data on immunohistochemical staining showed that the expression of JNK and caspase-12, two vital mediators in apoptosis, was reduced in myocytes of QX1-treated mice, compared with septic mice (**Figure 5**). Moreover, the overexpression of pro-inflammatory cytokine HMGB1 in septic mice myocytes was also reversed by treatment with QX1 (**Figure 5**). Considering the guiding significance of caspase-3, caspase-7, caspase-9, and caspase-12 in apoptosis evaluation, this study further examined the activation status of these indicators using Western blot analysis. The results demonstrated that septic mice subjected to CLP surgery displayed increased expression of cleaved caspase-3, caspase-9, and caspase-12 in myocytes (**Figure 6**). Nevertheless, QX1 treatment after CLP surgery markedly prevented the activation of caspase-3, caspase-9, and caspase-12. Although caspase-7 was also activated in cardiomyocytes of septic mice, QX1 treatment showed the limited regulatory effect on it (*p* > 0.05).

### QX1 Inhibited Apoptosis via ER- and Mitochondria-Associated Pathways in Myocytes of Septic Mice

**FIGURE 7:**
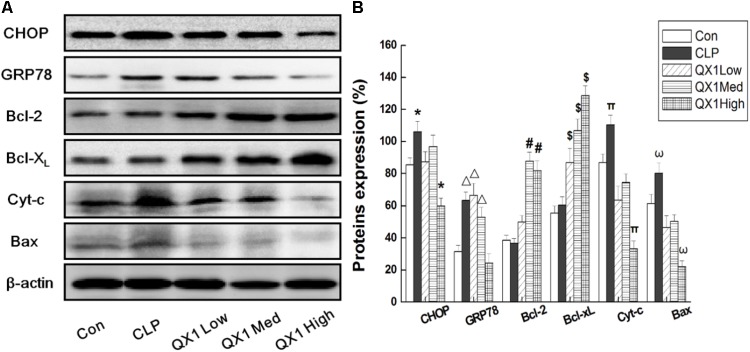
**(A)** QX1 inhibits apoptosis via ER- and Mitochondria-associated pathways in myocytes of septic mice. QX1 formula of three dosages (indicated as low dose, medium dose, and high dose, were 0.25, 0.5, and 1 g/kgbw, respectively) were orally administrated 2 h after surgery and heart tissue were obtained 48 h after CLP surgery for western blot detection. **(B)** Statistical analysis of indicated proteins expression. Data represent the mean ± SD of three independent experiments and differences between mean values were assessed by one-way ANOVA. ^∗^*p* < 0.05, ^Δ^ *p* < 0.05, ^#^*p* < 0.05, ^$^*p* < 0.05, ^π^*p*< 0.05, and ^ω^*p* < 0.05 indicate significant differences compared with control group.

On the basis of bioinformatic analysis and investigation on apoptotic indicators in myocytes, this study hypothesized that QX1 treatment alleviated the damages in septic heart mainly through mediating ER- and mitochondria-dependent signaling pathways. Therefore, key signaling molecules were examined to better understand the underlying mechanisms of the beneficial advantages of QX1. As shown in **Figure 7**, the expression of CHOP and GRP78, two important regulators in ER-associated apoptosis, was remarkably reduced in myocytes of QX1-treated mice compared with that in the CLP surgery mice. However, the expression of GRP94, another ER-associated apoptotic indicator, was not affected by QX1 treatment (data not shown). Moreover, QX1 treatment enhanced the expression of anti-apoptosis proteins (Bcl-2 and Bcl-X_L_) but suppressed pro-apoptosis protein (Bax and Cyt-c) generation, which might contribute to the inhibition of mitochondria-dependent apoptosis in septic heart.

## Discussion

Sepsis, recognized as a comprehensive and lethal syndrome caused by infection, contributes to excessive inflammatory response, immune dysfunction, and multiple-organ failure, making it one of the leading causes of mortality in ICUs (Angus and van der Poll, 2013). Although increasing number of studies have verified immunosuppression in the late stage as the primary reason of mortality, cytokine storm, and cardiac dysfunction in the early onset also lead to the death of septic patients (Hotchkiss et al., 2013; Prescott and Angus, 2018). A large number of studies have indicated that the reduction of heart damages contributes to better therapeutic efficacy and improved survival of septic patients (Hasslacher et al., 2011; Drosatos et al., 2013; Zhou et al., 2016; Rhodes et al., 2017). Traditional Chinese medicine has long been used for the clinical treatment of sepsis, some of which showed salutary therapeutic effects by improving cardiac functions. Many well-established reports have confirmed that active ingredients extracted from herbs exhibit promising therapeutic efficacy on SIC (Smeding et al., 2012; Zhai and Guo, 2016); moreover, traditional Chinese prescription, such as Xuebijing injection, has notable capacity in promoting M2 macrophage polarization and suppressing uncontrollable inflammatory response, thereby preventing heart damage in septic patients (Liu et al., 2015).

The QX1 formula is an effective prescription designed for treating congestive heart failure created by Professor Lijuan Huang, based on clinical practice in Beijing Hospital of Traditional Chinese Medicine for more than 30 years. This formula consists of five herbs, including Shui hong hua zi (*Polygonum orientale* L.) 30 g (3/8), Huang qi [*Astragalus membranaceus (Fisch.) Bge. var. mongholicus (Bge.) Hsiao*] 30 g (3/8), Fu ling 20 g [*Poria cocos (Schw.) Wolf*) (2/8), Dan shen (*Salvia miltiorrhiza Bge.*) 20 g (1/8), and Wu wei zi (*Schisandra chinensis (Turcz.) Baill.*] 10 g (1/8). QX1, through long-term clinical practice and multicenter studies, has been proved to be effective in heart failure by improving the clinical symptoms and prognosis of the patients. Recently, this study found that QX1 formula, as complementary therapeutic medicine for treating septic patients, obviously ameliorated the cardiac function and thus improved the curative effect and health outcomes (data unpublished). However, its potential regulatory property and underlying mechanisms have not been revealed yet. In this study, the composition of QX1 formula was evaluated. The UPLC–MS/MS analysis data of three formula samples showed that the ingredients of QX1 were stable and repeatable (the details of components are presented in Supplementary Data Sheet S2 and Supplementary Table S1). According to previous established studies, ingredients such as quercetin (Guo et al., 2017), tanshinone IIA (Yan et al., 2018), and daucosterol (Jiang et al., 2015) possess promising anti-apoptosis and anti-oxidant capacities via regulating vital signaling pathways.

This study, using *in vivo* survival data, proved that oral administration of QX1 effectively reduced the mortality of septic mice subjected to CLP surgery. Anatomical observation, together with histological evaluation of multiple organs, indicated that QX1 treatment ameliorated the structural and histopathological damages of septic mice, especially in the heart tissue. This study found a large number of inflammatory cells infiltrated in myocardial infarction area and obvious degeneration and necrosis at the edge in cardiac tissue of CLP mice. However, QX1 treatment notably decreased the number of infiltrated inflammatory cells and reduced necrosis and apoptosis in heart tissue. We also evaluated the regulatory effect of QX1 on the production of pro-inflammatory cytokines that have been reported to collectively contribute to SIC via decreasing the myocardial contractility (Liu et al., 2017). The results showed that QX1 treatment significantly decreased IL-1β secretion in plasma of septic mice 24 and 48 h after CLP surgery, exhibiting promising anti-inflammatory property. Moreover, although QX1 treatment also reduced TNF-α production 24 h after surgery, it showed limited effect 48 h after surgery. As a well-known anti-inflammatory cytokine, IL-10 was crucial in modulating excessive inflammatory response, whose high expression in early onset was beneficial to sepsis prognosis (Li et al., 2017). However, QX1 had little effect on IL-10 secretion in the present study. EF% and FS% in LV of mice are important indexes that reflecting the function of heart (He et al., 2018). Results based on echocardiography demonstrated that QX1 treatment bettered the function of septic mice by promoting EF% and FS% in LV.

This study employed genomics technology to provide an unbiased and overall assessment of gene expression profile to further understand the underlying mechanisms of QX1 on the cardiac tissue of septic mice. The data based on bioinformatic analysis showed that 647 genes in myocytes of QX1-treated septic mice were significantly regulated, of which 328 were upregulated and 319 downregulated, compared with septic mice without treatment. According to pathway enrichment analysis, significantly changed genes mainly enriched in signaling were related to cardiac diseases, including viral myocarditis, HCM, dilated cardiomyopathy, and so forth. Moreover, KEGG analysis illustrated that, compared with the genomic profile of myocytes in septic mice, QX1 treatment mainly affected pathways in the process of apoptosis, indicating that the expression of apoptotic genes coding caspase-3 and ATM was suppressed while that of anti-apoptotic genes, such as Bcl-2, Bcl-X_L_, IAP, and NF-κB, was enhanced. These data further prompted to investigate the role of QX1 on apoptosis in myocytes.

Current understanding suggests that intrinsic pathways that utilize ER and mitochondria are central to the process of apoptosis (Konstantinidis et al., 2012). Bcl-2 family proteins, including pro-apoptotic members (Bax and Bak) and anti-apoptotic members (Bcl-2 and Bcl-X_L_), contribute to mitochondria-dependent apoptosis. Meanwhile, ER stress–related activation of downstream mediators, such as CHOP, JNK, and caspase-12, induce apoptosis by enhancing the expression of downstream pro-apoptotic proteins such as Trb3 (Cheng et al., 2015) and Bim (Brouwer et al., 2017) and suppressing that of Bcl-2 (Vega et al., 2015). This novel study explored that QX1 treatment effectively prevented the activation of caspase family members (e.g., caspase-3, caspase-9, and caspase-12) in myocytes, which might contribute to its anti-apoptotic function. Considering the relative characters caspase proteins play in ER- and mitochondria-associated apoptosis, this study hypothesized that QX1 participated in both intrinsic apoptosis pathways. As expected, QX1 treatment suppressed ER-associated apoptosis by inhibiting the expression of CHOP and GRP78 in myocytes. Meanwhile, regulators of mitochondria-associated apoptosis, for instance, anti-apoptotic proteins (Bcl-2 and Bcl-x_L_) and pro-apoptotic ones (Bax and Cyt-c), were also regulated by QX1 treatment. By promoting the activation of anti-apoptotic proteins and preventing that of pro-apoptotic mediators, QX1 exhibited a promising therapeutic effect on apoptosis and damage, further contributing to improved outcomes of septic mice.

Herbal therapy has been well-investigated in recent years, and mounting evidence proves that multiherb formulas, composed of a combination of more than two herbs, have shown promising curative effects in treating kinds of diseases. In conclusion, this study demonstrated that QX1 suppressed apoptosis and damage in myocytes of septic mice by suppressing ER- and mitochondria-associated apoptosis and thus ameliorated heart tissue damages. Consequently, the results might provide guidance for the optimization and rationalization of traditional Chinese medicine formula.

## Ethics Statement

This study was carried out in accordance with the recommendations of “Committee for the Care and Use of Experimental Animals at Beijing Institute of Traditional Chinese Medicine.” The protocol was approved by the “Beijing Institute of Traditional Chinese Medicine.”

## Author Contributions

XXL: substantial contributions to the conception and design of the work; drafting the work and revising it critically for important intellectual content. LQQ: substantial contributions to the design of the work; interpretation of data for the work. HSS, ZJX, and WN: acquisition, analysis, and interpretation of data for the work. HXY: drafting the work and revising it critically for important intellectual content. GYH: substantial contributions to the design of the work; final approval of the version to be published; agreement to be accountable for all aspects of the work in ensuring that questions related to the accuracy and integrity of any part of the work are appropriately investigated and resolved.

## Conflict of Interest Statement

The authors declare that the research was conducted in the absence of any commercial or financial relationships that could be construed as a potential conflict of interest.
