# Supplementary material for: Qiang-Xin 1 Formula Prevents Sepsis-Induced Apoptosis in Murine Cardiomyocytes by Suppressing Endoplasmic Reticulum- and Mitochondria-Associated Pathways
Source: Front Pharmacol. 2018 Jul 30;9:818. doi: 10.3389/fphar.2018.00818 (PMC6077999; doi:10.3389/fphar.2018.00818)
Supplement: Supplementary file 1 [file Table_1.DOCX]

| Name | ID | Content (μg/mL) |
| --- | --- | --- |
| Gallic acid | Sample 1  Sample2  Sample3 | 22.7536  24.9056  21.0576 |
| Cryptotanshinone | Sample 1  Sample2  Sample3 | 58.2184  60.0392  51.6592 |
| Formononetin | Sample 1  Sample2  Sample3 | 2.1288  4.2032  6.8624 |
| Kaempferol | Sample 1  Sample2  Sample3 | 2.932  2.7576  3.5472 |
| Quercetin | Sample 1  Sample2  Sample3 | 15.5136  32.1304  39.5072 |
| Tanshinone IIA | Sample 1  Sample2  Sample3 | 125.748  102.3488  89.7464 |
| Texifolin | Sample 1  Sample2  Sample3 | 31.692  40.7344  41.6992 |
| Danshensu-sodium | Sample 1  Sample2  Sample3 | 40.0952  69.0472  75.8976 |

**Supplementary 3**

**Content of dominating compounds**

Ion chromatogram of QX1 Formula (3 samples)

**QX1 formula, Sample1, Positive ion flow**

**QX1 formula, Sample2, Positive ion flow**

**QX1 formula, Sample3, Positive ion flow**

**QX1 formula, Sample1, Negative ion flow**

**QX1 formula, Sample2, Negative ion flow**

**QX1 formula, Sample3, Negative ion flow**

**Chromatogram of dominating compounds**

**Gallic acid**

**Standard**

**Sample**

**Cryptotanshinone**

**Standard**

**Sample**

**Formononetin**

**Standard**

**Sample**

**Kaempferol**

**Standard**

**Sample**

**Quercetin**

**Standard**

**Sample**

**Tanshinone IIA**

**Standard**

**Sample**

**Texifolin**

**Standard**

**Sample**

**Danshensu-sodium**

**Standard**

**Sample**
