# Supplementary material for: Qiang-Xin 1 Formula Prevents Sepsis-Induced Apoptosis in Murine Cardiomyocytes by Suppressing Endoplasmic Reticulum- and Mitochondria-Associated Pathways
Source: Front Pharmacol. 2018 Jul 30;9:818. doi: 10.3389/fphar.2018.00818 (PMC6077999; doi:10.3389/fphar.2018.00818)
Supplement: Supplementary file 2 [file Table_2.DOCX]

**Supplementary 4**

**7-day secretion of cytokines**



**Fig. S2** ELISA detection on expression of serum cytokins (7 days after sugery).
