# Supplementary material for: Qiang-Xin 1 Formula Prevents Sepsis-Induced Apoptosis in Murine Cardiomyocytes by Suppressing Endoplasmic Reticulum- and Mitochondria-Associated Pathways
Source: Front Pharmacol. 2018 Jul 30;9:818. doi: 10.3389/fphar.2018.00818 (PMC6077999; doi:10.3389/fphar.2018.00818)
Supplement: Supplementary file 3 [file Data_Sheet_1.DOCX]

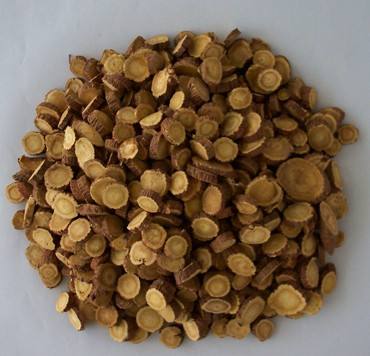


黄芪, Huang qi, *Astragalus membranaceus (Fisch.) Bge. var. mongholicus (Bge.) Hsiao*

Obtained from the Inner Mongolia Province, China.


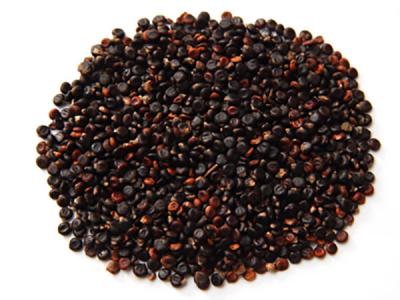


水红花子，Shui hong hua zi (*Polygonum orientale L.*)

Obtained from the Heilongjiang Province, China.


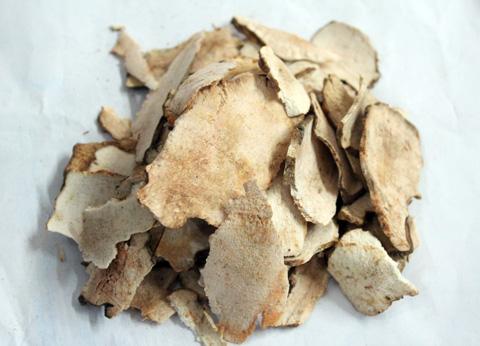


茯苓, Fu ling, *Poria cocos (Schw.) Wolf*

Obtained from the Yunnan Province, China.


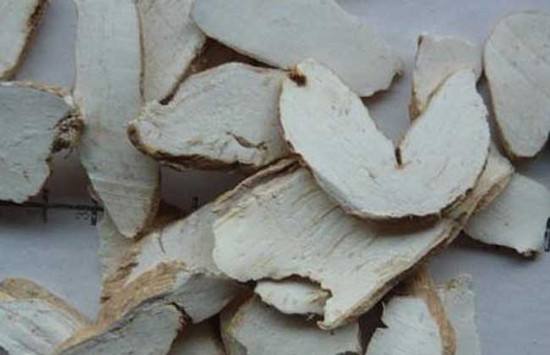


丹参, Dan shen, *Salvia miltiorrhiza Bge.*

Obtained from the Hebei Province, China.


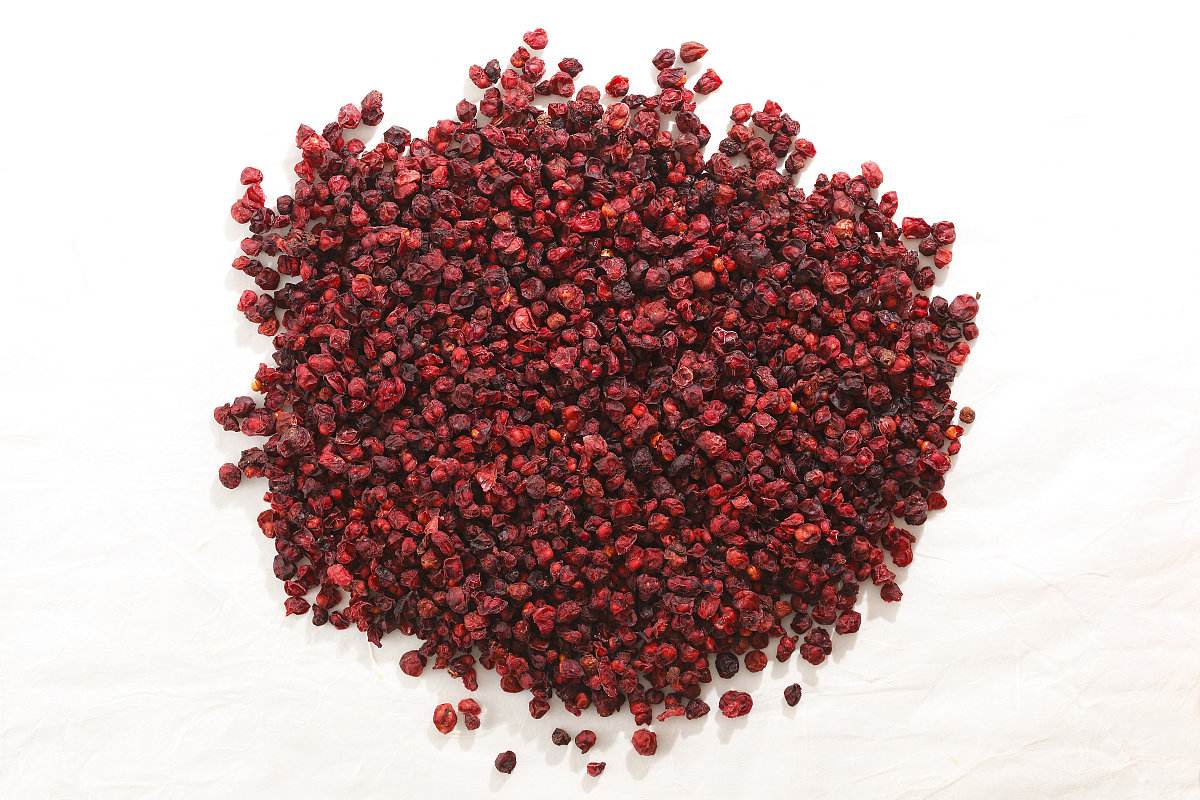


五味子，Wu wei zi, *Schisandra chinensis (Turcz.) Baill.*

Obtained from the Liaoning Province, China.
