# Supplementary material for: Qiang-Xin 1 Formula Prevents Sepsis-Induced Apoptosis in Murine Cardiomyocytes by Suppressing Endoplasmic Reticulum- and Mitochondria-Associated Pathways
Source: Front Pharmacol. 2018 Jul 30;9:818. doi: 10.3389/fphar.2018.00818 (PMC6077999; doi:10.3389/fphar.2018.00818)
Supplement: Supplementary file 4 [file Data_Sheet_2.DOCX]

**Composition of QX1 formula**

**kadsuric acid**

**gomlsin B**

**gomisin G**

**trimethyl citrate**

**myristic acid**

**daucosterol**

**gomisin j**

**chamigrenal**

**gomisin a gomisin f**

**nigranoic acid**

**gomisin k1**

**tigloylgomisin p**

**tetracosanoic acid**

**epigomisin o**

**poricoic acid e**

**eburicoic acid**

**ergosterol**

**tumulosic acid**

**undecanoic acid**

**dehydropachymic acid**

**β-amyrin acetate**

**dehydrotumulosic acid**

**oleanolic acid**

**trametenolic acid**

**p-Hydroxyphenylethanol ferulate**

**naringenin**

**5,7-dihydroxychromone**

**sitosterol**

**dihydroquercetin**

**quercitrin**

**Isoquercitrin**

**betaine**

**linolic acid**

**astragaline F**

**kaempferol**

**calycosin**

**ferulic acid**

**polycanthisine**

**choline**

**folic acid**

**quercetin**

**formononetin**

**ferruginol**

**Tanshinone I**

**Tanshinone IIa**

**Dihydrotanshinone I**

**Ruscogenin**

**Calycosin**

**Calycosin-7-glucoside**

**Panoxatriol**

**deoxyschizardrin**

**Schisandrin B**

**Cryptotanshinone**

**Protopanaxadiol**

**Ferulic acid**

** Tanshinol**

**Salvianolic acid A**

**p-Coumaric acid**

**Quercetin**

**Hyperoside**

**Caffeic acid**
